# Tubo-Ovarian Abscess Occurring 16 Years After Supracervical Hysterectomy

**DOI:** 10.1080/10647440300025516

**Published:** 2003

**Authors:** Toshimitsu Tohya, Toshihiro Yoshimura, Chikashi Onoda

**Affiliations:** Department of Obstetrics and Gynecology Kumamoto Rosai Hospital Yatsushiro Kumamoto 866-8533 Japan

## Abstract

*Background:* Supracervical hysterectomy is seldom performed and there are few reports of tubo-ovarian abscess (TOA) after supracervical hysterectomy.

*Case:* The case of a 49-year-old woman with a right TOA is reported. This patient had received a supracervical hysterectomy 16 years earlier due to rupture of the uterus. At this admission, she presented with complaints of lower abdominal pain and fever. Bimanual and transvaginal ultrasound examinations demonstrated a tender mass in the right adnexal region. Laparotomy, pathologic examination and microbiologic study confirmed the diagnosis of right TOA.

*Conclusion:* After supracervical hysterectomy, patients may develop endocervicitis, parametritis and/or TOA. This series may be a subtype of ascending infections in the female genital tract.


*Infect Dis Obstet Gynecol 2003;11:167–169*

### Tubo-ovarian abscess occurring 16 years after supracervical hysterectomy

**Toshimitsu Tohya, Toshihiro Yoshimura and Chikashi Onoda**

*Department of Obstetrics and Gynecology, Kumamoto Rosai Hospital, Yatsushiro, Kumamoto, Japan*

***Background:*** Supracervical hysterectomy is seldom performed and there are few reports of tubo-ovarian abscess (TOA) after supracervical hysterectomy.
***Case:*** The case of a 49-year-old woman with a right TOA is reported. This patient had received a supracervical hysterectomy 16 years earlier due to rupture of the uterus. At this admission, she presented with complaints of lower abdominal pain and fever. Bimanual and transvaginal ultrasound examinations demonstrated a tender mass in the right adnexal region. Laparotomy, pathologic examination and microbiologic study confirmed the diagnosis of right TOA.
***Conclusion:*** After supracervical hysterectomy, patients may develop endocervicitis, parametritis and/or TOA. This series may be a subtype of ascending infections in the female genital tract.

Key words: OVARIAN ABSCESS; PELVIC INFLAMMATORY DISEASE; HYSTERECTOMY

### INTRODUCTION

Pelvic inflammatory disease (PID) is caused by microorganisms that colonize the endocervix ascending to the endometrium and fallopian tubes, and tubo-ovarian abscess (TOA) is an end-stage process of acute PID. We report the pathophysiology of a case of TOA in a patient who had undergone supracervical hysterectomy 16 years earlier.

### CASE REPORT

A 49-year-old woman, gravida-3, para-2, presented at our clinic complaining of lower abdominal pain and chills in April 1999. The patient had undergone a supracervical hysterectomy in July 1983 because of uterine rupture. From 5 days before this admission, the patient had been treated with a 5-day course of intravenous cefoperazone (1 g two times a day) at another medical clinic, for fever of unknown origin. Physical examination at that time revealed an axillar temperature of 38.6°C, blood pressure of 120/80 mmHg, and pulse rate of 78 bpm. A CT scan at the other hospital revealed a lower abdominal mass. She was referred to our hospital. Physical examination revealed localized mild tenderness in the right lower abdominal quadrant. On bimanual examination, the right adnexa were tender, cystic in texture and hardly fixed. Movement of the uterine cervix resulted in considerable pain. Specular examination revealed a creamy vaginal discharge. The cervix was strongly displaced to the left by the right adnexal mass. Ultrasound examination demonstrated that the mass was cystic and measured 54 × 62 × 64 mm in size. A CT scan demonstrated that the lesion was

Correspondence to: Toshimitsu Tohya, MD, Department of Obstetrics and Gynecology, Kumamoto Rosai Hospital, Yatsushiro, Kumamoto, 866-8533, Japan. Email: tohya@fine.ocn.ne.jp

© 2003 The Parthenon Publishing Group 

nonhomogeneous, which became more obvious after an intravenous injection of contrast material made the central, low-density region of the mass more evident. The initial laboratory evaluation at the previous clinic revealed that the white blood cell count was 12 200/μl, hemoglobin concentration was 11.3 g/dl, C-reactive protein was 5.7 mg/dl and serum electrolytes and chemistry were almost normal. Urinalysis was also normal. Initial evaluation at our hospital demonstrated a white blood cell count of 8200/μl and an axillar temperature of 36.4°C. Therefore, conservative antibiotic therapy (clarithromycin 200 mg two times a day) was started, but she gradually developed fever and lower abdominal pains. Because of persistent abdominal pain, a laparotomy was performed on her 5th hospital day. There was a small amount of pus in the pelvic cavity when the peritoneum was opened. Examination of the pelvic organs revealed the presence of moderately marked adhesions of the ileum and omentum from Douglas pouch to the right pelvic wall. Dissection of the above adhesion revealed a ruptured right TOA, a normal-sized left adnexa adhering to the left pelvic wall and the intact appendix. The peritoneum over the residual cervix was gritty and slightly reddish. No diverticula were observed on the small and large intestine. Bilateral salpingo-oophorectomy with transabdominal drainage was performed. The pus culture grew *Bacteroides ovatus* and *Streptococcus milleri*. The pathological report demonstrated acute inflammation involving the right fallopian tube and ovary. She received antibiotic therapy (cefepime 1 g two times a day for 7 days). Postoperative progress was uneventful. She was discharged on postoperative day 14.

### DISCUSSION

Tubo-ovarian abscess after supracervical hysterectomy is very rare. Total abdominal hysterectomy or vaginal hysterectomy is commonly performed. Despite the high frequency of abdominal or vaginal hysterectomy, TOA usually does not develop postoperatively, and only exceptional cases of TOA 4–6 years after hysterectomy have been reported[1–4]. Usually, the main route of TOA infection involves an ascending mechanism, although other routes (hematogenous, lymphatic, direct etc.) are possible. Supracervical hysterectomy is performed only in very rare cases, such as uterine rupture at the time of Cesarean section. However, the pathophysiology of TOA after supracervical hysterectomy is not clear. Therefore, the route of infection of this case of TOA should be considered.

The pathophysiology of bacterial infection in this case differs from a purulent inflammatory process secondary to the passage of bacteria from the uterine cavity into the tubal lumen and the ovary. Instead, TOA formation may include seeding via hematogenous infection, diverticular disease or appendicitis. Infection extending from another nearby organ was not likely in this patient because there was no evidence of diverticulitis or appendicitis. Hematogenous infection is much less likely apart from exceptional cases, such as tuberculosis. However, there was no evidence of tuberculosis infection found in this case.

The beginning of this infection probably lay in a deterioration of vaginal self-cleaning. Primarily, bacterial vaginosis may have occurred. This was previously referred to as nonspecific vaginitis and is an alteration of normal vaginal bacterial flora that results in the loss of lactobacilli and an overgrowth of predominantly anaerobic bacteria. The alteration of vaginal flora facilitates the ascending spread of pathogenic bacteria by enzymatically altering the cervical mucus barrier.

The cervix is made up of two different types of epithelial cells: squamous epithelium and glandular epithelium. *Neisseria gonorrhoeae* and *Chlamydia trachomatis* infect only the glandular epithelium and are responsible for mucopurulent endocervicitis[5]. Mucopurulent endocervicitis is characterized by purulent endocervical discharge[6], which was not observed when the diagnosis of TOA was established.

During supracervical hysterectomy, the uterine cervix is closed by suturing the cervix and peritonization is performed. However, the sutured cervix may necrotize and the peritoneum may be the only barrier against ascending bacterial infection. Therefore, microorganisms colonizing the endocervix may ascend and induce TOA. In our case, the uterine cervix was sensitive for several days postoperatively. Therefore, we think that cervicitis and parametritis occurred first, then TOA formed. Further, the site of the infection demonstrated polymicrobial infection with a high prevalence of anaerobic organisms. TOA can occur in patients who have undergone supracervical hysterectomy through an ascending route, similar to that in a woman who has a uterus.

### REFERENCES

1. Mendez LE, Bhoola SM, Horowitz JR. Bilateral tubo-ovarian abscess four years after total hysterectomy. *Infect Dis Obstet Gynecol* 1998;6:138–40
2. Lau M, Cross CA, Berens P, *et al*. Ovarian abscess 15 months after vaginal hysterectomy. A case report. *J Reprod Med* 1997;42:669–71
3. McCausland VM, Fields GA, McCausland AM, Townsend DE. Tuboovarian abscesses after operative hysteroscopy. *J Reprod Med* 1993;38:198–200
4. Hueston WJ. A case of tubo-ovarian abscess 6 years after hysterectomy. *J Ky Med Assoc* 1992;182: 399–402
5. Kiviat NB, Paavonen JA, Wolner-Hanssen P, *et al*. Histopathology of endocervical infection caused by *Chlamydia trachomatis*, herpes simplex virus, *Trichomonas vaginalis*, and *Neisseria gonorrhoeae*. *Hum Pathol* 1990;21:831–7
6. Brunham RC, Paavonen J, Stevens CE, *et al*. Mucopurulent cervicitis – the ignored counterpart in women of urethritis in men. *N Engl J Med* 1984;311:1–6

RECEIVED 02/19/03; ACCEPTED 07/16/03
